# Power, autonomy, and the construction of the ‘difficult patient’ in the labor ward: Insights from maternal health providers in western Kenya and northern Ghana

**DOI:** 10.1371/journal.pgph.0007069

**Published:** 2026-11-13

**Authors:** Joyceline Kinyua, Edwina N. Oboke, Monica Getahun, Beryl A. Ogolla, Irene Kuwolamo, John Baptist K. Dorzie, Ali Moro, Raymond A. Aborigo, Linnet Ongeri, Patience A. Afulani

**Affiliations:** 1 Center for Virus Research, Kenya Medical Research Institute, Nairobi, Kenya; 2 Research Department, Global Programs for Research and Training, Nairobi, Kenya; 3 Department of Obstetrics, Gynecology and Reproductive Sciences, University of California, San Francisco, California, United States of America; 4 Social Science and Public Health Department, Navrongo Health Research Center, Navrongo, Ghana; 5 Department of Epidemiology and Biostatistics, University of Technology and Applied Sciences, Navrongo, Ghana; 6 Center for Clinical Research, Kenya Medical Research Institute, Nairobi, Kenya; 7 Department of Epidemiology and Biostatistics, University of California, San Francisco, San Francisco, California, United States of America; NYU Grossman School of Medicine: New York University School of Medicine, UNITED STATES OF AMERICA

## Abstract

Disrespect and abuse during childbirth are frequently justified by providers on the grounds that the woman was “difficult.” Although challenging patient-provider interactions have been recognized as a driver of poor maternity care, little is known about how providers construct the notion of the “difficult patient” or how they navigate these situations. We explored maternity care providers’ perspectives on factors that contribute to difficult situations during childbirth and on their responses to such encounters in Kenya and Ghana. We conducted 51 in-depth interviews with maternity care providers, including nurses, midwives, doctors, clinical officers, and support staff, in western Kenya (n = 31) and northern Ghana (n = 20). Interviews were audio-recorded, transcribed verbatim, and analyzed using codebook thematic analysis with a combination of deductive and inductive coding. Providers described difficult situations as arising from the interaction of patient, provider, and health system factors rather than from patient characteristics alone. Four themes contributed to these situations: patients’ attitudes, behavior, and psychological state; conflicting beliefs and preferences; encounters in which healthcare providers were patients; and challenging workplace conditions. Providers responded by seeking assistance from colleagues or family members, adapting communication strategies, enforcing clinical instructions, temporarily disengaging from difficult interactions, or respecting patients’ wishes while documenting refusals or referring patients when necessary. While some responses aligned with principles of person-centered care, others reflected tensions between respecting patient autonomy and maintaining provider authority under resource-constrained conditions. Our findings challenge the notion of the inherently “difficult patient” by demonstrating that challenging maternity care encounters are socially and structurally produced through interactions among women, providers, and the health system. Interventions to improve respectful maternity care should address not only provider communication and interpersonal skills but also the organizational conditions and power dynamics that shape patient-provider interactions during childbirth.

## Introduction

Improving maternal health remains a global priority, given plateaus in progress towards reducing maternal mortality [1]. An estimated 700 women continue to die daily from preventable pregnancy and childbirth-related complications, with low- and middle-income countries (LMICs), particularly in sub-Saharan Africa (SSA), bearing a disproportionate burden [1]. Despite substantial progress in increasing access to facility-based childbirth services, the maternal mortality ratio (MMR) in SSA remains high at 454 per 100,000 live births, accounting for 70% of the global maternal deaths [1]. Over 80% of births in Ghana and Kenya occur in health facilities [2,3], yet their MMRs remain high at 370 per 100,000 live births in Kenya and 234 per 100,000 live births in Ghana [1]. These statistics point to significant gaps in the quality of care, highlighted in the Lancet Commission on High-Quality Health Systems report [4].

High-quality care is effective, efficient, timely, safe, person-centered, and equitable [5]. Quality of care encompasses both technical and interpersonal aspects, and the World Health Organization (WHO) recommendations emphasize the provision of evidence-based services and a positive childbirth experience [6,7]. Yet studies continue to document negative care experiences during childbirth, including disrespect and abuse from providers and facilities [8,9]. In Kenya and Ghana, studies have reported that women experience different forms of disrespect and abuse, including non-dignified care such as physical, verbal, and emotional abuse, neglect, non-consented care, poor communication, and lack of support [10–15]. Several factors account for these negative experiences, but a common reason providers cite for mistreating certain patients is that the patient was “difficult” [13,16–19].

The “difficult patient” label has long been used in the medical literature to describe patients who are medically or interpersonally challenging. This includes patient behaviors deemed “undesirable,” such as uncooperativeness, demanding, ingratitude, unfriendliness, noncompliance, disrespect for providers’ authority, and immorality [16,20–24]. Providers tend to seek clarity, order, and control, and thus often prefer patients who agree with them and let them be in charge, thereby rewarding acquiescence by calling some patients “good” and others “difficult” [21,25]. The practice of labeling patients as “difficult” has been criticized for implying that the problem lies primarily with the patient. However, evidence suggests that such perceptions arise from a complex interaction of patient characteristics and behaviors, provider attitudes and expectations, and the broader healthcare environment. Consequently, the term “difficult situations” has been proposed as a more appropriate and less stigmatizing alternative, as it recognizes the multifaceted nature of challenging clinical encounters [21].

Maternity wards in low-resource settings are a fertile context for such difficult situations. Women in labor may experience unmet physical, emotional, and informational needs, while providers may hold rigid expectations regarding appropriate patient behavior during childbirth. These dynamics are further compounded by understaffing, inadequate resources, high workloads, and provider stress and demotivation, creating conditions that can strain patient-provider interactions and contribute to negative care experiences [16].

Although difficult situations have been identified in several studies as the driver of disrespect and abuse, they have not been explored in detail in the maternal health literature. Our study, therefore, sought to explore maternal health providers’ perspectives on difficult situations encountered during facility-based childbirth. Specifically, we asked two research questions: (1) What factors contribute to difficult situations in maternity care? and (2) how do providers respond to them? Understanding how providers experience and respond to difficult situations will help identify opportunities to improve both provider support and women’s experiences of care.

## Methods

### Ethics statement

We obtained ethical approval from the office for the Protection of Human Subjects at the University of California, San Francisco (IRB number 17–22783); the Kenya Medical Research Institute Scientific and Ethics Review Unit (SERU 3682); and the Navrongo Health Research Center Institutional Review Board (NHRCIRB386). We further sought permission from the Kenya National Commission for Science, Technology & Innovation (NACOSTI) and the Migori County Director of Health in Kenya; and the Upper East Regional Director of Health Services and the District Directors of Health in Ghana. We obtained written informed consents from all healthcare providers to participate in the study.

### Study design, setting, and research participants

This study was part of the formative research for the Caring for Providers to Improve Patient Experience (CPIPE) intervention, which employed a convergent mixed-methods study design with maternal healthcare providers in Migori County, western Kenya, and the Upper East Region (UER), northern Ghana [26]. The quantitative data and other findings are published elsewhere [27–32]. In this paper, we used a descriptive qualitative approach to explore providers’ perspectives on the factors contributing to difficult situations and how these situations were addressed. The sites were selected because they represent high-burden maternal mortality regions and have been previously described [27–32]. Migori County has about 315 facilities, including one County referral hospital, eight sub-county hospitals, and several lower-level facilities, such as health centers and dispensaries [33]. The UER has one regional referral hospital, 11 district/municipal hospitals, 67 health centers, and ~419 Community-Based Health Planning and Services (CHPS) compounds [34]. Clinical and non-clinical health providers working in maternity units at high-volume delivery facilities in the study sites of both countries were eligible to participate. We conducted interviews with 51 providers: 31 providers from 20 facilities in Kenya and 20 providers from 13 facilities in Ghana.

### Data collection procedures

The data collection procedures are described in detail elsewhere [27–32] and summarized here. Facilities were eligible if they had at least 50 births per month. The facilities were ranked, and those with the highest births in the county/region were selected. With permission letters from the county/regional directorate, the research assistants visited the selected health facilities. They introduced the study to the facility and maternity heads, who provided a list of all maternity providers, from which we purposefully sampled and recruited. We aimed for at least one doctor or clinical officer (if any), two or more nurses (depending on the number available), and one or two support staff members to ensure variation across the different provider cadres in the maternity unit. But at several facilities, particularly in Kenya, all providers in the maternity unit who were available on the days of the interview were invited to participate, as fewer providers were available than the targeted number for those facilities.

In both cases, providers were first informed about the study, and their consent to participate in the survey was obtained. At the end of the survey, they were asked whether they would be willing to participate in in-depth interviews. A purposive sample was then selected from those who consented to the in-depth interviews (aiming for at least one from each facility and variation in provider cadre), and a Research Assistant (RA) followed up to schedule the interviews. Four RAs trained in social science and qualitative data collection methods (two based in Kenya and two in Ghana) conducted the interviews in English using a semi-structured interview guide (see supplemental file S1 Text). The interviews lasted from 40 to 60 minutes. The interviews were conducted in Kenya from 15th June to 27th September 2019 and in Ghana from 25 January to 29 March 2021 (the time interval between the interviews in Kenya and Ghana reflects the different implementation periods in the two countries, with delays in Ghana due to the COVID-19 pandemic). All interviews were audio-recorded and transcribed verbatim.

### Data analysis

We used the codebook thematic analysis approach to identify themes related to providers’ perceptions of factors contributing to difficult situations and how they dealt with them [35]. Data analysis combined deductive and inductive approaches. An initial coding framework was developed based on the study objectives and relevant literature. As transcripts were reviewed, additional codes were generated inductively from the data and incorporated into the coding framework. The coding framework was then iteratively refined during coding of the initial transcripts before being applied to the remaining transcripts (see supplemental file S2 Text). After coding all transcripts, we generated code reports that included all text segments associated with each code and systematically reviewed them. We grouped related codes into broader themes, summarized relevant text excerpts to characterize each theme, and selected illustrative quotes to represent participants’ perspectives. Theme development was an iterative process, involving regular discussions among the research team to review emerging interpretations and ensure consistency in the analysis. Themes and subthemes were further refined during the writing process to provide a clear and accurate representation of participants’ experiences and views. Themes were identified based on their salience and explanatory power rather than frequency [36].

### Reflexivity

Our team consists of researchers from SSA, based in Kenya, Ghana, and the US, with expertise in qualitative research and in the social sciences, public health, and medicine. Our approach to the data collection, analysis, and interpretation recognizes our positionality as SSA researchers, including those in the diaspora. We recognize how our identities shape the questions we ask and how we interpret the data. We view this as a strength of our approach: one that leverages local knowledge and expertise, prioritizes engaging those who collected the data throughout the full research cycle, and fosters discussion rather than seeking objectivity or conformity. Two Kenyan-based RAs [EO and BO] and two Ghanaian-based RAs [IK and JB] conducted the interviews and contributed to the analysis and interpretation of the data. The remaining team members contributed to other research activities, including data analysis and interpretation, under the lead investigator’s [PA] oversight.

### Inclusivity in global research

Additional information regarding the ethical, cultural, and scientific considerations specific to inclusivity in global research is included in the Supporting Information (S2 Checklist)

## Findings

### Characteristics of the study participants

About half of the participants in both countries worked in government hospitals, with the rest working in health centers and private hospitals. Most were nurses/midwives, with a more diverse group of providers in Kenya, including clinical officers/doctors, and support staff. Most had less than 11 years of experience. In Ghana, all providers were female, whereas in Kenya, two-thirds were female. Most providers in both countries were married and between 30 and 40 years old. See distributions in Table 1.

**Table 1:** Participant characteristics.

	Kenya (N = 31)		Ghana (N = 20)	
	No.	%	No.	%
**Facility**				
Gov’t Hospital	18	58.1	11	55.0
Gov’t Health Centre/Dispensary	9	29.0	5	25.0
Mission/Private Hospital	4	12.9	4	20.0
**Cadre/position**				
Nurse/Midwife	18	58.1	19	95.0
Clinical officer/Doctor	3	9.7	0	0.0
Support staff	10	32.3	1	5.0
**Years as provider**				
0–5 years	15	48.4	9	45.0
6–10 years	11	35.5	5	25.0
≥ 11 years	5	16.1	6	30.0
**Gender**				
Female	21	67.7	20	100.0
Male	10	32.3	0	0.0
**Age**				
< 30	10	32.3	6	30.0
30–40	14	45.2	12	60.0
≥ 40	7	22.6	2	10.0
**Marital Status**				
Married	23	74.2	15	75.0
Single/divorced/widowed	8	25.8	5	25.0

### Factors that contribute to difficult situations

We identified four themes that capture providers’ perceptions of factors contributing to difficult situations in the maternity units. These themes included: patients’ attitudes, behaviors, and psychological states; conflicting beliefs and preferences; providers as patients; and challenging workplace conditions. Of note, during the interviews, providers referred to the people they cared for as patients, clients, mothers, or women. Thus, these terms are maintained in participant quotes. In the analysis, we use ‘women’ with biological processes specific to pregnancy and birth and ‘patients’ in general descriptions to align with the literature on difficult-patient-provider interactions.

#### Patients’ attitudes, behaviors, and psychological state.

Four attitudes and behaviors of patients—uncooperative, disrespectful, the use of herbal medications, and presenting late in labor—in addition to the patient’s emotional and psychological state, were described as leading to difficult situations:

Difficult situations often arose when a patient was deemed uncooperative, which led to challenging interactions with the provider. Such situations included instances in which patients doubted the information they were provided during care and disregarded provider instructions.

*“……Some mothers, when the nurse has examined them and tells them they are not ready to deliver that day, start saying the nurse does not know what she is doing. So it makes us, the staff, to just look at the mother because she seems to know more than us…” (SS1081, Ghana)*

*“Then again, the patients themselves, just like we have said, there are some who are uncooperative, so maybe the attitude that they give you will also determine how you handle them…” (CP1221, Kenya)*

Patients who declined examinations were also considered uncooperative, which led to difficult situations. Providers noted that vaginal examination during labor is critical for clinical decision-making. Yet, some women resisted it, which was perceived as complicating the pre-birth procedures.

“*…… we explain to them the procedure that goes on in the labor room, the time we come to examine them, to reexamine or to recheck whether everything is moving on well. You explain all this, yet you get to someone, and she tells you she doesn’t want someone to touch her, she doesn’t want you to put your fingers in the vagina, yet the examination is very important in evaluating labor progress.” (CP1206, Ghana)*

“*Yes, some patients…for example, taking a vaginal examination... some of them do not allow.”* (*CP2012*, Ghana)

Providers became frustrated when patients did not follow their instructions or behaved in a manner they perceived was against the expected behavior during labor, especially if they perceived the behavior could result in a poor birth outcome.

*“Now I get frustrated because I feel like I can see this child and the fetal heart rate is fine, but it’s declining because this client is not cooperating, so now, I get irritated…”* (CP1171, Ghana)

Providers recognized that patients were often uncooperative when they did not understand what they were told. Thus, it was important to take time to explain things to them. But they noted that some patients remained uncooperative even after understanding what was happening. They were thus especially frustrated when patients remained uncooperative after they had taken the time to explain. Providers justified the ‘difficult’ patient label, citing persistent resistance despite education.

“*In everything, you have to assure the patients and tell them your findings. For instance, when a patient is in labor, and you ask her to do something to help her or not to do something, some patients will not mind you at all. Let’s say she is 4cm or 5cm, and she starts to push, and I ask her not to push because it is not yet up, they will not even mind you. They will not listen to whatever advice you are giving them, and they will do what they want. So, I will call such people difficult...” CP0113, Ghana)*

*“…When the mother does not cooperate with you, you say this, and the person does otherwise. For example, someone is not fully dilated to push the baby out, but she is pushing. You give the person the necessary education and the implications of what the person is doing, but the person still does the same thing; you can say the person is difficult. Especially when the person understands what you are saying, yet still does the same thing, but if the person does not understand, we can say it’s because the person doesn’t understand you. But if the person understands you, the communication between you is fine, everything is fine, yet she still does the same thing, we can say the person is difficult.” (CP0816, Ghana)*

Support staff also reported encountering difficult situations where patients and their families were unwilling to do what was expected of them: for instance, not stepping on the floor while cleaning is in progress. In addition, some indicated that they have worked in the maternity ward for a long time and have developed some knowledge of the birthing process. However, when dealing with patients and their family members, they are generally looked down upon as lacking expertise because they are cleaners, which they don’t like.

*“You can find that you are telling a mother who has danger signs that there is a possibility of being taken for an operation (CS) to save her and her baby, she ignores and sees like you are disturbing her, so I usually feel so bad and wish that I had a way in which I could even just save the unborn child…” (SS1102, Kenya)*

Patients’ disrespect also led to difficult situations. Disrespect was described in terms of actions, communication, or attitudes patients displayed toward providers, which the providers perceived as challenging their authority and hindering the smooth provision of services. For instance, patients who considered themselves to belong to a higher socio-economic status were said to sometimes interfere with service provision by making demands. They noted this happened because they believed that their financial status justified preferential treatment and that they should be treated differently from the rest.

*“They believe that they are really rich, and they are expecting special treatment from you, so as much as you try to explain to them that everyone is equal here, they don’t want to listen to you, so you just keep quiet…” (CP1081, Kenya)*

Other patient behaviors leading to difficult situations included taking herbal medications from traditional birth attendants (TBAs) in the local community, which were believed to hasten labor and cause the woman to begin pushing before she was fully dilated. This often led to complications, at which time they then presented at the health facility. When they arrived at the facility, it complicated the type of care they could offer them.

“*This area is chaotic for traditional birth attendants; they start these herbs that, in their opinion, hasten the labor, and they only refer to the facility when they think that there is a problem, and they can’t handle any more. The herbs I hear that they are meant to work like oxytocin, you also want to put in oxytocin, which is an intervention for you, so it’s like you are doubling the dose, and you are risking tears or even rupturing the uterus, so that affects, it depends on how they have come...” (CP1172, Kenya)*

“*She came and examined her, and the mother was pushing with strong contractions, so we suspected that she had drunk the concoction from the TBA, so the doctor tried to talk to her not to push.” (CP1181, Kenya)*

Further, when women labored at home and presented very late, it created a difficult situation as providers did not have time to prepare for the birth. Sometimes, providers had to assist with such deliveries outside the labor ward, which was unsafe.

*“That mother had been laboring at home, and all these times she was laboring to herself, so I think when it was last minute, they jumped on a motorbike…. but just at the door of the maternity, the mother was delivering. I was barehanded; I didn’t have anything. So, I told a subordinate to bring me some gloves because that mother just started delivering at the door. It was not a very good picture for me seeing a mother deliver at that place, but I struggled to deliver that baby and took the mother in, so that was not very good…” (CP1222, Kenya)*

Providers noted that multiparous women were more likely to have had a previous experience with the TBAs. As such, they are likely to present in the hospital only when they have sensed a difficult birth, and usually, they are difficult to deal with since they already feel they can give birth without the support of the providers.

*“Multi (...Meaning having more than one birth…), not most of them, but some disturb. She feels like she has given birth before, like two to three times. More so if you find, like, the previous births, she went to TBA… Yeah, she feels like she can give birth without being assisted, and when you see her come to the hospital, there could be something because the hospitals are different from TBA, but multis are hard to deal with, sometimes...”* (CP2022, Kenya)

The labor period was said to cause a temporary but extreme discomfort, which affected women’s emotional and psychological state. This could be complicated by prolonged labor, leading to instances where some women exhibited temporarily aggressive behavior, which they felt ashamed of after they had given birth. Other times, they ignored whatever the providers told them, which made their management difficult.

*“Such situations exist in the facility because even some patients, if she has gone into labor for a long time, you find that they even tear the curtains and drop them down, so you just leave her to do all that she does, then when she is through, you just go and take them back. Later, you tell her how she was behaving, then she apologizes and seeks forgiveness by saying it’s only the labor that was making her behave in that manner. Because during labor she can even get hold of a chair, can end up breaking it, but after labor, she tells you, ‘sorry sister’” (SS2072, Kenya)*

#### Conflicting beliefs and preferences.

Some providers also noted that certain patient beliefs led to difficult situations. For instance, some religious beliefs prevent female patients from being attended by male healthcare providers. This is especially problematic when there is a staff shortage and a male provider is on duty.

*“Also, some refuse, like male nurses cannot attend to them, and this is especially with the Muslims, and yet some duties are only for males. So, it becomes difficult because we have to rotate in all the departments...” (CP2019, Kenya)*

Patient’s preference regarding who may attend to them during childbirth was also noted as a cause of difficult situations. This included a preference based on the provider’s gender or age. Others preferred specific providers, which might pose challenges when those providers were unavailable.

*“Yes, we have gender issues… sometimes the patients feel like they can’t be attended to by a man, and it happens very much with the clinical officer (mentions his name). When they come here to deliver, they will tell you that they want a female nurse; she is the one who will attend to me. Some have faith in a particular doctor, so when they find him or her away, then they don’t cooperate...” (CP2011, Kenya)*

In addition, some patients perceived young providers as less qualified, and sometimes conflated body size with age. Since healthcare provision should not be discriminatory, these situations were deemed difficult situations, especially when patients refused to cooperate because they preferred a different provider.

“*They have this perception, in a way that maybe somebody might not feel like, like we people who have small sized-bodies like we cannot help them(women) …. laughing… the old mothers can even ask you that, ‘young man, what do you know, we have seen a lot, and so you can’t explain to us anything’….” (CP2011, Kenya)*

#### Providers as patients.

Providers mentioned that fellow healthcare providers who came to give birth created difficult situations, especially when they repeatedly suggested clinical procedures to those attending to them or contradicted their clinical findings. As a result, some providers preferred not to attend to their colleagues who came in labor, citing professional role conflict, where the boundaries of authority are blurred.

*“Some…. Like these fellow health providers, I don’t like providing services to most of them. Some I have treated so well until later, after I have finished attending to them, is when I realize that, so this is a nurse just like me. But some will tell you so many things that you will just say that, ‘this is a health care provider’. She will use the medical terms on you, as she will ask you, ‘Is this meconium?’” (CP1181, Kenya)*

*“Yes, very stressful when she is a healthcare provider, she will say I have not dilated. I have not felt like I am having contractions and have not done what…” (*CP2022, Kenya)

#### Challenging workplace conditions.

Providers acknowledged that structural factors, such as high workload and insufficient resources, can cause stress and lead them to act with hostility toward patients. The stress of a high workload was said to particularly affect communication with patients, leading to difficult situations.

*“When the workload is so much, you are so stressed up, sometimes communicating to clients is a bit difficult, you are not able to give out your best….and when the systems too are not working at the hospital, for example, you need that, and you are not getting it, you need oxygen, you are not getting it, it makes the work a bit frustrating.” (CP1417, Ghana)*

*“I think it’s still based on stress and pressure…You know if you are stressed …and then someone is asking you unnecessary questions, and giving you more pressure, you can just get angry and talk to the person anyhow. But if you had enough rest and go to work, no matter how the person provokes you, you’ll even laugh over it because you are not stressed.” (CP0715, Ghana)*

Patients may, in turn, not want to be attended to by such providers, given that they do not understand the prevailing circumstances. This inability to understand one another leads to a difficult interpersonal situation between the patient and the provider.

*“When you are stressed at work, you can approach a woman in a way that will make her angry, which you wouldn’t know. But because they can’t approach us, they will go to the house and talk about what you did to them.” (SS0114, Ghana)*

The work environment often interacts with other factors, such as women’s conflicting beliefs, attitudes, and behavior, to produce difficult situations. For instance, because they did not have certain resources, such as extra linens, available, they usually advised women during antenatal clinic visits on items to bring during delivery as part of birth preparedness. Some may, however, show up in labor without such items because preparing for birth contradicts their cultural belief, leading to challenging situations.

*“…sometimes they are too difficult, like when you explain to them somethings, they don’t do that. Like when... at the maternal clinic, we tell them that when they are nearing thirty-six weeks, they should always walk with at least two ‘lessos’ (Linen), identify a birth companion, and some little cash, because labor can get them anywhere. But since they believe it’s a taboo to prepare anything for an unborn child, a mother will come with the only clothe that she has on her body. Like in our facility, we don’t have the linens for our beds, so you will struggle, or you are forced to use that mothers’ clothes to wipe the baby and this can result in other infections to the baby. They don’t understand, and so at times it forces you to become a little bit harsh to save the baby.” (CP5032, Kenya)*

Working in a facility without adequate capabilities for managing certain complications also led to difficult situations when mothers refused referrals to higher-level facilities.

*“Like you have managed a mother, and it is now prolonged labor, the child has fetal distress, you have resuscitated, and now you want to refer, and they don’t agree, you have to be tough on them. They feel like this is a hospital, and where you are taking them is also a hospital, and so they prefer that they remain in this facility, and they don’t understand that we don’t admit patients in our facility. All they know is that if there is a doctor and a nurse in a facility, then it is complete. Like if you tell them that this child needs observation or even the mother needs to be observed. So in such a case, you just call an ambulance and refer.” (CP10257, Kenya)*

### Provider response to difficult situations

Difficult situations evoked various feelings in providers, which they described using terms like ‘not good,’ ‘bad,’ ‘unhappy,’ ‘sad,’ ‘stressed,’ ‘mad,’ ‘angry,’ ‘unstable,’ ‘irritated,’ ‘unappreciated,’ ‘frustrated,’ and ‘torturing.’ These feelings influenced how they responded. We identified five themes describing how providers responded to difficult situations: asking for assistance, improving provider communication, enforcing provider instructions, ignoring the patient, and respecting patient wishes.

#### Asking for assistance.

A common approach to dealing with difficult situations was to ask for help. Providers reported that when a patient refused to take their advice, they turned to their colleagues, especially the more experienced ones, for help. This was recognition that the situation stemmed from the patient’s perceptions of the specific provider caring for them. They thus empathized with the patient and sought assistance to help them.

*“I really feel bad, but again, besides feeling bad, I will have a feeling that there must be a reason, maybe they are not comfortable with me handling them. Number two, maybe they don’t have trust in me, or maybe these people, the way I talk, the perception they have of me, could be the reason why they don’t want to cooperate. But for me, if I learn that the patient is not comfortable with me, I will feel bad and ask myself if I have done something wrong. But to me, it’s not a big issue, so I will refer that patient to another provider. (CP2011, Kenya)*

*“… I try to talk to colleagues … we discuss and sometimes find solutions to it. Or talk to a senior colleague because probably, the way you handled it wasn’t the best, and you can handle it another way…” (CP0816, Ghana).*

Other times, they asked family members for help, especially when patients refused to comply with instructions at crucial moments when they deemed cooperation was important to preserve the life of the unborn baby. They noted that it can be quite annoying and frustrating when they ‘have to’ use force on a patient, so they usually call the family members to help, and to observe how their family member is behaving, so that they are not blamed when death occurs. Of note, some of these family members also used forceful and abusive behaviors to gain compliance, as in the scenario below.

*“Yes, even the mother-in-law talked to her; she just killed her baby like that. It was a big struggle. She was twisting my hands while I was trying to help her remove the baby. What helped me is that I am big in size, and she was small; that is what saved my arm from a dislocation. People came, and the husband slapped her…she even twisted my hand as if she wanted to break it, it is only that I am big, I managed to remove my hands, had it been that I was slender, it would have been a dislocation. But people came, some got hold of her hands, some pressed her down, then the husband came, slapped her, and then the baby came out.” (SS2021, Kenya)*

*“That is why initially I said you will use all the techniques, you beg, sometimes you involve the family to come and talk to them, especially when there is someone related to them. You engage the person, let the person come in…” (CP1206, Ghana)*

Some providers, however, noted that when they realized that an accompanying relative might aggravate the situation, they requested that the relative leave, especially if the patient could express themselves.

*“I will remove the family member and continue to deal with the client because she is the one who can decide on her health” (CP2043, Ghana)*

#### Improving provider communication.

As noted previously, providers recognized that difficult situations often resulted from ineffective communication in which the patient and provider did not understand each other. Thus, a response mentioned was improving their communication. For instance, language barriers and cultural differences may affect communication between the provider and the patient, requiring a different approach, such as using practical examples, speaking the local language, requesting a translation, or speaking at a level the patient is likely to understand, in the hope that using a language the patient understands better would foster cooperation.

*“I can use the language that she best understands, like if she is a (mentions tribe), then I explain to her in (the local dialect), giving her my example of what happened to me, so when I went through the same thing that you are being told, I got helped, so please just cooperate and be helped.” (SS1102, Kenya).*

*“I don’t actually think we have difficult patients; I think it has to do with the understanding. When someone finds it difficult or is not able to understand you, people conclude that they are difficult. But if you are able to come down to the person’s level, speak with patience, she will understand.” (CP0715, Ghana)*

Improving communication often came from a place of empathy, putting themselves in the patients’ shoes, exercising patience, and trying to do their best despite the situation.

*“… it’s only that it’s the language, when the person is not good, you don’t have to become annoyed too. So sometimes you put yourself in their shoes; they are human, so it’s normal. But as time goes on, they will change and calm down.” (CP2501, Ghana)*

*“You see, for humans, there is a saying that there is no action without a cause.” Maybe they might have a reason for not cooperating. But when I try explaining to them, most of the time, when they themselves see that the benefit they stand to gain from what we are doing outweighs whatever reason they have, they end there.” (CP0507, Ghana)*

Providers noted that, even when it was annoying and frustrating, they tried to suppress their anger, avoid arguing with the patient, and talk to them calmly.

“*When I see them like that, I just continue to talk to them without really getting angry. I assume that maybe she doesn’t know me, so I just introduce myself to them and explain to them softly until they understand me” (SS1111, Kenya).*

*“I just talk to them and pretend that I am not angry because they are the ones who have created employment for us, sometimes when she is too much, then I just go and talk to the nurse to talk to her” … (SS2131, Kenya)*

#### Ignoring the patient.

Providers sometimes ignored patients they perceived as difficult. Especially in moments of extreme frustration or high workload, some providers opted to withdraw interaction to prevent escalation.

*“So when they are rude, and maybe they don’t like those instructions, you end up frustrated because that is the right thing you will have to do when you cannot do them, then you simply document and remain there staring and watch…” (CP1222, Kenya)*

*“You know, as a human being, you have to deal with this thing,… because they are supposed to behave like that…. [so] you understand, and leave them alone.” (SS1012, Ghana).*

For some, ignoring the patient was often a last resort after they had explained, tried to persuade, pampered, and called others for help.

*“I just kept quiet. After all I’ve explained everything to you, I will just keep quiet and tell you that with all that you are doing, don’t go and say somewhere that its my fault, because I’ve done my possible best; I’ve explained, pampered you because with the nature of our work, we know how the pain is so we pamper you and sometimes we even call some of the clients relatives who have gone through it before to talk to you. If after all these, the person is still misbehaving, I will just keep quiet, since I’ve already told you all that I have to, and when the outcome comes, you will have to take it like that.” (CP0801, Ghana)*

#### Enforcing provider instructions.

Some providers noted they quietly enforce their instructions, such as insisting on administering the correct medication even when the patient declined it, and ignoring the patient’s preferences. While others said they used forceful means to put pressure on the patient to comply.

“*Like when a woman comes, and she even wants to tell me where to inject. I tell them why I need to inject this position, and not the other that she wants me to. Because with them, they think that anywhere is injectable, ‘last month you injected me at this point, so this time round please don’t, you can only inject here.’ So, after explaining to her and she does not listen, I simply do the right thing. You know you can’t listen to the wrong thing that a woman is saying, so what I mostly do is a cold war, I simply keep quiet, and I just work quietly.” (CP1181 Kenya)*

*“It is a little bit frustrating, but we still probe further once we know the danger signs ahead. We try as much as possible to put a little pressure.” (CP2101, Ghana)*

A few noted that they sometimes got angry when, after all their effort, including begging, the patient refused to comply, and raised their voice at the patient. But they were quick to say that although this sometimes worked in getting the patient to comply, it was only occasional.

*“Sometimes it’s very frustrating. But as a professional, you have been trained, so if it involves begging, you do beg. We just plead that they just understand with us and comply. But at the worst will be to raise your voice, which mostly doesn’t occur.” (CP1206, Ghana)*

*“You know, as human beings, though we are trained to be professionals in our line of duty, but as we are human beings and as young as we are, sometimes the anger comes, but when we display our anger, it sometimes keeps them calm. Then, they would realize that this person means business, so let me go back and wait. So, when we display our anger, it works sometimes, but doesn’t work other times because it’s not everybody that you would even open your eyes to, and they would succumb to it.” (C 0210, Ghana)*

#### Respecting patient wishes.

A final response to difficult situations was to respect the patient’s wishes while taking steps to avoid negative consequences for the provider. This included documentation to prevent the possibility of negligence charges. Providers noted that when patients declined care or behaved in ways they believed could lead to poor outcomes, they documented the situation to protect themselves from potential consequences from the patient’s behavior.

*“Sometimes, with several minutes or hours of talking, others don’t want to be examined, so you only write in the folder, patient’s right. The person declined all explanations. We don’t force them, it’s their right…” (CP1206, Ghana)*

Some providers felt unappreciated when patients were uncooperative or disrespectful, because they felt their best efforts were not valued. But they noted they continued to do their best for them.

*“It’s like your work is not appreciated. What you are doing to them, they don’t see any impact. Anything you say, they will do the other way round. You will feel you are not appreciated. Because you will try to do your best for the patient, but the patient will never cooperate. But you will continue to do the best for the person.” (CP0661, Kenya)*

Others said they referred such patients to another facility to get them off their hands and avoid the potential negative consequences. Referral was often a last resort after they had tried other strategies.

*“If she is still not comfortable, then you refer because this is one patient whom you can decide to inject, then she reacts, and she again says that you injected her with bad intentions even though you did not have bad intentions while you were giving her injections…”(CP2012, Ghana)*

*“It is usually not easy. When it happens that way, you have to exercise patience and talk to the person. If the person is still not understanding, we have to refer the client to be handled at the next level.” (CP0419, Ghana)*

## Discussion

We explored maternity providers’ perspectives on factors contributing to difficult situations during facility-based childbirth in Kenya and Ghana and how they respond to these situations. Providers attributed difficult situations to a combination of patients’ perceived attitudes and behaviors and the emotional and psychological effects of labor. They also cited conflicting beliefs and preferences, encounters in which healthcare providers were patients, and structural constraints within health facilities. Their responses ranged from seeking support and adapting communication strategies to ignoring patients, enforcing compliance, and respecting patient wishes while documenting refusals or referring women elsewhere. These findings support critiques of the difficult-patient label, which obscures the relational and contextual nature of challenging encounters by locating the problem primarily within the patient. The findings contribute to the growing literature on respectful maternity care by illuminating how providers interpret and navigate challenging interactions and how these may shape care experiences.

Consistent with prior literature on the “difficult patient,” providers frequently described patients as difficult when they questioned clinical advice, declined examinations, challenged provider authority, or failed to follow instructions during labor [20,25]. Providers often expect patients to follow instructions during labor without questioning and thus interpret deviations from these expectations as problematic. Yet, childbirth presents a unique context in which women experience severe pain, fear, vulnerability, and uncertainty, which, coupled with stressed providers working under challenging conditions, creates the perfect storm for difficult situations [16,28]. Providers themselves acknowledged that many behaviors they found challenging emerged during prolonged or painful labor and often resolved after birth. Thus, many of the behaviors described by providers as leading to difficult situations are attempts by women to cope with pain and fear, maintain bodily autonomy, or assert control in a setting where they often have limited power [37,38]. Women who first went to traditional birth attendants, where they were given herbal medications that were perceived to interfere with medications given during labor, and those who presented late in labor challenged providers’ ability to care for them appropriately. Herbal medicine use is common in Sub-Saharan Africa and has been cited as a common practice among pregnant women [39]. This raises a broader question of why some women may choose to seek care from traditional birth attendants as their first point of contact during labor; reasons are often related to mistreatment in health facilities, and have been described in detail elsewhere [14,40–42].

The findings also reveal how power operates in maternity care. Compliance is often used as a marker of the “good” patient, and resistance as evidence of difficulty. Such expectations reflect longstanding hierarchies within biomedical care that position providers as authority figures and patients as passive recipients of care [25]. Childbirth is, however, recognized as a period where women seek not only safe clinical care but also dignity, autonomy, and respect for their preferences [7]. Women’s refusal of examinations, requests for specific providers, or questioning of clinical recommendations can therefore be understood as efforts to exercise agency rather than as simple acts of noncompliance. Participants’ perceptions of colleague-patients as particularly difficult because they questioned clinical decisions, used medical terminology, and challenged assessments further support providers’ need to maintain the hierarchy. Healthcare providers who become patients may feel empowered to participate actively in decision-making, whereas providers may be accustomed to a more directive model of care. Provider discomfort thus stems not only from patient behaviors but also from disruptions to traditional power hierarchies in clinical encounters. The tension between provider authority and women’s autonomy, evident in our findings, reflects broader struggles over control and decision-making documented in maternity care globally [43–45].

Additionally, the findings underscore the importance of understanding difficult situations within the realities of under-resourced health systems. Preferences regarding provider gender, age, or specific clinicians were often viewed as barriers to the provision of care. These, however, represent legitimate expressions of dignity, privacy, and cultural values. Provider responses, thus, undermined person-centered principles of respect for patient preferences because staffing shortages limited that flexibility.

Of note, providers rarely attributed their behavior to the difficult situation. Still, they linked their behavior that led to challenging encounters to stressful working conditions due to inadequate resources and high workloads. These findings align with research demonstrating that provider burnout, emotional exhaustion, and workplace stress are associated with poorer patient-provider interactions and increased risk of disrespectful care [16,29,46]. In contexts where providers are expected to manage large caseloads with limited resources, maintaining patience, empathy, and respectful communication can become increasingly difficult. Thus, although individual provider attitudes play a role, difficult situations cannot be understood solely through the lens of provider and patient behavior but must be situated within broader organizational and structural conditions. “Difficult patients” are the products of difficult situations arising from the intersection of individual behaviors, social expectations, power relations, and health system constraints [21,22], reinforcing the growing recognition that efforts to improve respectful maternity care must address not only provider behavior but also the broader health system conditions in which care is delivered.

The strategies providers described for managing difficult situations revealed both promising practices and areas of concern. Providers described several constructive responses to difficult situations that align with principles of person-centered care and respectful maternity care [7]. Seeking assistance from colleagues, adapting communication to women’s linguistic and cultural contexts, appropriately involving trusted family members, attempting to remain calm despite frustration, and respecting preferences demonstrate awareness of strategies to respect and preserve patient autonomy while managing clinical risks. Descriptions of suppressing frustration, remaining calm despite provocation, and continuing to communicate respectfully even when they felt angry or challenged, on the other hand, represent forms of emotional labor: the management of emotions to fulfill professional expectations [47,48]. While these strategies may help preserve therapeutic relationships, emotional labor can become burdensome when performed continuously under stressful conditions and without adequate organizational support. Over time, the strain associated with managing difficult interactions may contribute to emotional exhaustion, depersonalization, and burnout, creating conditions that undermine respectful care.

Further, providers described responding to difficult situations by ignoring women, overriding their preferences, or enforcing compliance when they believed women were making poor decisions. In some cases, family members were enlisted to secure cooperation, occasionally through coercive or physically forceful means. While providers often justified these actions as necessary to protect maternal or fetal well-being, such practices blur the boundary between beneficent care and coercion. They illustrate how unequal power relations can become normalized within childbirth care, particularly when providers perceive women’s actions as threatening clinical outcomes. This, in turn, led to instances of overt obstetric violence and mistreatment, including verbal and physical abuse of women, which have been previously documented [13,16,17,49].

Finally, maternity providers’ responses to difficult situations also involved navigating tensions between respecting women’s choices and ensuring what they perceived as safe clinical outcomes. These varied responses, ranging from persuasion, referral, documentation, selective engagement, and enforcement of instructions, are best understood through the lens of street-level bureaucracy, which emphasizes how frontline workers exercise discretion to manage uncertainty when implementing policies under conditions of limited resources and competing demands [50,51]. From this perspective, difficult situations are not merely interpersonal conflicts but moments in which providers negotiate competing institutional expectations, professional responsibilities, and resource constraints. Such negotiations may sometimes result in practices that compromise person-centered care, even when providers are motivated by concern for patient well-being.

The findings have important implications for policy, practice, and future research. First, interventions to improve respectful maternity care should explicitly address difficult situations and equip providers with practical skills, effective communication, shared decision-making, conflict resolution, de-escalation, and trauma-informed care. Second, provider training should encourage reflection on how assumptions about compliance, authority, and appropriate patient behavior shape interactions with women during childbirth. Third, health systems must address structural factors that contribute to difficult situations, including staffing shortages, high workloads, inadequate supplies, and provider burnout. There is also a need for holistic approaches that address the well-being of both patients and providers. Further, antenatal education and community engagement initiatives may help improve communication between women and providers by addressing expectations surrounding labor and birth. Finally, research into the impact of such interventions is needed.

This study has limitations. The findings reflect providers’ perspectives and do not capture patients’ interpretations of the same encounters. Future research should examine difficult situations from both provider and patient perspectives to better understand how perceptions of difficulty are constructed and negotiated. Providers largely framed difficult situations as external to their own practice, potentially reflecting social desirability bias. They also provided constructive approaches to dealing with difficult situations, without directly mentioning disrespect or abuse as a response, as documented in prior studies. In addition, the study was conducted in specific regions of Kenya and Ghana, which may limit generalizability to other contexts. The sample was also imbalanced by gender, with all providers in Ghana being female. Thus, it may not be representative of the views of male maternity providers. The sample distribution, however, reflects the gendered nature of the maternal health workforce. The study was not intended to be a comparative, but similar themes emerged across both samples. The inclusion of providers from two countries and multiple facility types thus strengthens the relevance of the findings and highlights common patterns across settings.

In conclusion, providers in Kenya and Ghana viewed difficult situations during childbirth as arising from a complex interplay of patient behaviors, emotional responses to labor, sociocultural preferences, provider-patient dynamics, and health system constraints. While providers often framed these situations as involving difficult patients, the findings suggest that many challenges are relational and contextual rather than attributable solely to the patient. Efforts to improve respectful maternity care should move beyond a focus on individual behaviors to addressing the structural and relational conditions that shape interactions during childbirth. Supporting providers in managing difficult situations by improving working conditions, providing communication training, and implementing mechanisms to address burnout is critical to fostering more respectful, equitable, and person-centered maternity care.

## Supporting information

S1 Checklist COREQ checklist. (PDF)

S2 Checklist PLOS inclusivity in global research questionnaire. (DOCX)

S1 Text Interview guide. (DOCX)

S2 Text Coding framework. (DOCX)
