# Supplementary material for: Power, autonomy, and the construction of the ‘difficult patient’ in the labor ward: Insights from maternal health providers in western Kenya and northern Ghana
Source: PLOS Glob Public Health. 2026 Nov 13;6(11):e0007069. doi: 10.1371/journal.pgph.0007069 (PMC13766216; doi:10.1371/journal.pgph.0007069)
Supplement: Supplementary file 3 [file pgph.0007069.s003.docx]

Provider in-depth interviews

DATE____________	District NAME__________________________	FACILITY NAME: ____________________________	RESPONDENT ID____________

**SWITCH ON TAPE RECORDER**

**My name is ________________ I am with respondent number ________ in ___________________district on _____________(date)**

| **Main Questions** | **Specific probes** | **General probes** | **Comments** |
| --- | --- | --- | --- |
|  |  |  |  |
| 1. Tell me a little about yourself | Besides your work as a ______ How do you enjoy spending your time? | a. Can you tell me more about that? b. What actually happened? c. Can you walk me through what happened, step by step? d. Can you give me an example e. What makes you say that? f. How did that make you feel? |  |
| 2. Tell me how a typical work day looks like for you |  |  |  |
| 3. Give me an example of one of the best days you have ever had at work |  |  |  |
| 4. Give me an example of one of the worst days you have ever had work |  |  |  |
| 5. Have you ever experienced anything (else) that you will describe as traumatic? | How did you deal with that situation or similar situations emotionally? |  |  |
| 6. What are some of the challenges of working in your position in this facility? | What makes you most satisfied or happy about your job?  What makes you most frustrated or sad about your job? |  |  |
| 7. What stresses you? | How do you feel about the amount of effort you put into your work compared to the rewards you get from it?  How do you feel when you think you are stressed out? |  |  |
| 8. What do you do when you are feeling stressed or burnt out? | What do you think can be done to help you deal better with stress or burn out? |  |  |
| 9. What are some of the things that affect how you interact with patients? | How do you feel when a woman or her family is not cooperating with you?  What will make you say a patient is difficult?  What do you do when a woman or her family is rude or not following instructions?  What makes it worse?  What makes it better?  How do you deal with it? | a. Can you tell me more about that? b. What actually happened? c. Can you walk me through what happened, step by step? d. Can you give me an example e. What makes you say that? f. How did that make you feel? |  |
| 10. Patients report that sometimes they are treated differently based on a number of factors. What do you think contributes to differential treatment? | Are there certain things that can trigger the way patients are treated?  Are some patients less likely to cooperate?  Are there particular types of patients you prefer to care for?  Are there particular types of patients you don’t like to care for? |  |  |
| 11. Have you ever participated in a training in how to improve your interactions with patients? | If yes: Did you find it helpful?  What did you find helpful about it?  Why did you not find it helpful?  If no: Will you like to take part in such a training?  What will you like to be included in such a training? |  |  |
| 12. Have you ever taken part in a training on how to reduce stress? | Did you find it helpful?  What did you find helpful about it?  Why did you not find it helpful?  Will you like to take part in a training to help reduce stress?  What will you like to be included in such a training? |  |  |
| 13. What formats of training will you like? | Will you prefer group or individual sessions? Why do you say that?  Who will you like included in your group? For example, will you like a training that includes doctors, nurses, midwives, and support staff in the same group? Why do you say so?  Will you like a training that involves the use of an app downloaded to your phone? Why do you say so?  How long do you think the training should be? Why do you say so? |  |  |
| 14. What type of support can help improve your work experience? | What do you think about when you hear mentorship?  Do you have a mentor?  If no will you like one?  What will you like from a mentorship relationship?  Will you like to mentor someone?  Will you like to be in a peer support group? Why or why not |  |  |
| This is the end of our interview. Thank you so much for taking the time to answer our questions.  Do you have any questions for me about this interview?  What was it like for you to participate in this interview? | Is there any other thing you think we should know that I have not asked about? |  |  |

Summary Impressions
