# Supplementary material for: Power, autonomy, and the construction of the ‘difficult patient’ in the labor ward: Insights from maternal health providers in western Kenya and northern Ghana
Source: PLOS Glob Public Health. 2026 Nov 13;6(11):e0007069. doi: 10.1371/journal.pgph.0007069 (PMC13766216; doi:10.1371/journal.pgph.0007069)
Supplement: Supplementary file 4 [file pgph.0007069.s004.docx]

**Coding Framework**

*Codes, sub-codes and definitions*

| **No.** | **Code** | **Definition** |
| --- | --- | --- |
| **1** | **Leisure activities** | Activities the provider describes doing outside of their clinical duties, usually in response to questions about how they spend their free time. Apply the relevant sub-code for each activity mentioned. |
| **1.1** | **Go out** | Provider describes going out socially for enjoyment, such as going to dance, drink, or visit bars or clubs. |
| **1.2** | **Go to church** | Provider describes attending church or taking part in other religious services or activities in their free time. |
| **1.3** | **Going to work** | Provider describes work (at the facility or another job) as a main way they spend their time, or says they have little time for anything outside work. |
| **1.4** | **House work** | Provider describes household chores and domestic responsibilities, including cooking, cleaning, caring for the home and farming. |
| **1.5** | **Interacting with others** | Provider describes spending free time socialising or interacting with friends, neighbours, colleagues or community members (excluding family, see 1.10). |
| **1.6** | **Listen to music or radio** | Provider describes listening to music or the radio, or singing, as a leisure activity. |
| **1.7** | **Movies and TV** | Provider describes watching movies, television programmes or other video content in their free time. |
| **1.8** | **Reading** | Provider describes reading (e.g., books, the Bible, newspapers, study materials) as a leisure activity. |
| **1.9** | **Run a business** | Provider describes running or working on a business or other income-generating activity outside their clinical job. |
| **1.10** | **Spending time with family** | Provider describes spending free time with their spouse, children or other relatives. |
| **2** | **About provider** | Discussions of the provider's personal and professional background in response to introductory or ice-breaker questions, such as "Tell me a little about yourself" and "Besides your work as a ______, how do you enjoy spending your time?" Includes cadre, years of experience, education, family situation and current role. |
| **3** | **Challenges** | Descriptions of difficulties the provider faces in their role. These may relate to the facility, patients, other providers or the wider health system. Can be double coded with Causes of stress (14.1). |
| **3.1** | **Delayed or inadequate salaries** | Provider describes salaries that are paid late, not paid, or too low for the work they do. |
| **3.2** | **Difficulties with referrals** | Provider describes problems referring patients to other facilities or levels of care, such as unavailable receiving facilities, delays, poor communication or patients refusing referral. |
| **3.3** | **Experience not valued by other providers** | Provider feels their knowledge, skills or experience are not respected by other providers, including feeling that colleagues or superiors do not listen to them. |
| **3.4** | **Inadequate staff/High patient loads** | Provider describes too few staff for the number of patients, leading to heavy workloads, long shifts or providers covering multiple roles. |
| **3.5** | **Lack of drugs, equipment, and supplies** | Provider describes shortages or stock-outs of medicines, medical equipment or consumable supplies needed to deliver care. |
| **3.6** | **Patient attitude and behavior** | Provider describes patient behaviours or attitudes that make their work difficult, such as being uncooperative, rude, not following instructions or arriving late. |
| **3.7** | **Providers selling supplies to patients** | Provider describes other providers selling facility drugs or supplies to patients that should be provided free or through official channels. |
| **3.8** | **Transportation issues** | Provider describes transport problems affecting care, including their own travel to work, patients reaching the facility, or transporting patients for referral (e.g., lack of ambulances). |
| **4** | **Dealing with difficult patients** | Strategies the provider describes using when interacting with patients (or their families) whom they find difficult. Apply the relevant sub-code for each strategy mentioned. |
| **4.1** | **Ask colleagues to help** | Provider asks a colleague or superior to step in, assist, or take over care of a difficult patient. |
| **4.2** | **Ask family member to leave** | Provider asks a family member or companion to leave the room or ward because they are disruptive or making care more difficult. |
| **4.3** | **Ask family to help** | Provider asks the patient's family members or companions to help calm, persuade or support the patient. |
| **4.4** | **Ask if they want to be cared by someone else** | Provider offers the patient the option of being cared for by another provider. |
| **4.5** | **Document** | Provider records the patient's behaviour, refusal of care or the incident in the patient's notes or facility records, often for accountability or self-protection. |
| **4.6** | **Leave them alone** | Provider leaves the patient alone for a period, giving them space or time to calm down before continuing care. |
| **4.7** | **Not arguing with them** | Provider deliberately avoids arguing or reacting with the patient, remaining calm and patient. |
| **4.8** | **Use language they understand** | Provider communicates in the patient's own language or in simple terms the patient can understand in order to gain their cooperation. |
| **5** | **Difficult to classify** | Use for text that does not clearly fit any existing code. These excerpts will be reviewed later and recoded if needed. |
| **6** | **Feedback about interview/study** | Any comments by the participant about taking part in the interview or study, including their views on the questions, the process or the value of the research. |
| **7** | **Going the extra mile** | Provider describes going beyond their required duties to help patients or colleagues, such as using their own money or resources, staying after their shift, or doing extra tasks to ensure good care. |
| **8** | **Great quotes** | Particularly clear, powerful or illustrative quotes that capture a key issue and may be used in reports or publications. Always double code with the relevant content code. |
| **9** | **Impact of stress** | Provider descriptions of how stress affects them, their patients and other people around them. Apply the sub-code for who is affected. |
| **9.1** | **Others** | Effects of the provider's stress on people other than themselves and their patients, including colleagues, other staff at the facility, and family or others at home. |
| **9.2** | **Patients** | Effects of the provider's stress on their patients or on how they care for patients. Apply the relevant sub-code where possible. |
| 9.2.1 | Irritated or annoyed with patients | Provider describes becoming irritated, short-tempered or annoyed with patients as a result of stress. |
| 9.2.2 | Negative attitudes | Provider describes developing negative attitudes towards patients (e.g., impatience, unfriendliness, rudeness, neglect) as a result of stress or difficult work conditions. |
| **9.3** | **Self** | Effects of stress on the provider themselves, emotionally, physically or in their personal life. Apply the relevant sub-code where possible. |
| 9.3.1 | Discouragement | Provider describes feeling discouraged or losing hope or confidence in their work because of stress. |
| 9.3.2 | Family relationships | Provider describes stress affecting their relationships with spouse, children or other family members (e.g., bringing stress home, less time or patience for family). |
| 9.3.3 | Feel sad, bad, or hurt | Provider describes feeling sad, bad, hurt or emotionally low because of stress. |
| 9.3.4 | Frustrated or annoyed | Provider describes feeling frustrated or annoyed as a result of stress. |
| 9.3.5 | Health issues | Provider describes physical or mental health problems they attribute to stress, such as headaches, high blood pressure, fatigue, poor sleep or illness. |
| 9.3.6 | Lack of motivation or demoralization | Provider describes losing motivation, interest or morale in their work because of stress. |
| 9.3.7 | Negative thoughts | Provider describes having negative or worrying thoughts because of stress (e.g., dwelling on bad outcomes, self-doubt). |
| 9.3.8 | Wanting to quit | Provider describes wanting or considering leaving their job, the facility or the profession because of stress. |
| **10** | **Other factors that impact patient care** | Factors, other than those coded elsewhere, that the provider says influence how they care for or interact with patients. May be double coded with Stress (14). |
| **10.1** | **Attitudes and behaviors of family members** | Attitudes and behaviours of patients' family members or companions that affect care, such as interfering, making demands, being supportive, or failing to provide required items. |
| **10.2** | **Explain** | Provider describes explaining procedures, conditions or instructions to patients, and how this affects the patient's cooperation or the quality of the interaction. |
| 10.2.1 | Not being appreciated and valued | Provider feels their efforts (including explaining care to patients) are not appreciated or valued by patients or others, and describes how this affects care. |
| **10.3** | **Facility level factors** | Characteristics of the facility that affect patient care, such as infrastructure, space, privacy, equipment, supplies, staffing and management. Can be double coded with Causes of stress (14.1). |
| **10.4** | **How provider initiates interaction or speak to patient** | How the provider begins an interaction or communicates with a patient, such as greeting and introducing themselves, tone of voice, and building rapport. |
| **10.5** | **How providers present themselves** | How the provider presents themselves to patients, including appearance, dress, demeanour, professionalism and confidence. |
| **10.6** | **Language barriers** | Difficulties in communication between provider and patient because they do not share a common language. |
| **10.7** | **No preferred patients** | Provider states that they treat all patients the same and do not have preferred patients. |
| **10.8** | **Non-work related stress** | Stress arising from outside work (e.g., personal, family or financial problems) that the provider says affects how they care for patients. |
| **10.9** | **Patient attitudes and behavior** | Descriptions of patient attitudes and behaviours that affect care, including patients being difficult, uncooperative, anxious, not understanding instructions, or being cooperative and appreciative. |
| **10.10** | **Perceptions about provider capability based on age** | Provider describes patients or others judging their competence based on their age (e.g., young providers being seen as inexperienced). |
| **10.11** | **Provider attitudes** | Discussions of how the provider's own attitudes (e.g., patience, empathy, respect, or negative attitudes) affect interactions with patients and the care provided. |
| **10.12** | **Requests to birth in alternative positions** | Provider describes patients asking to give birth in positions other than lying on the bed (e.g., squatting, kneeling, on the floor) and how this affects care. |
| **10.13** | **Role of media** | Provider describes the influence of the media (e.g., radio, TV, social media) on patients' expectations, knowledge or perceptions of providers, and how this affects care. |
| **10.14** | **Share personal experience** | Provider draws on or shares their own personal experiences (e.g., of pregnancy, birth or illness) and describes how these shape how they care for patients. |
| **10.15** | **Time spent with patients** | The amount of time the provider is able to spend with each patient, including giving patients enough time to discuss their concerns. |
| **10.16** | **Time women present** | The stage of labour or pregnancy at which women arrive at the facility (e.g., early, late, in advanced labour) and how this affects care. |
| **11** | **Patient considered difficult** | Characteristics or behaviours that lead providers to see a patient as difficult, particularly regarding how the patient responds to the care or treatment provided. |
| **11.1** | **Disrespectful to providers** | Patient is seen as difficult because she is disrespectful to providers or looks down on them. |
| **11.2** | **Prolonged/severe labour** | Patient is seen as difficult because her labour is prolonged or severe, making her harder to manage. |
| **11.3** | **Providers negative attitude towards a patient** | Provider acknowledges that a patient may be labelled difficult because of the provider's own negative attitude towards her, including unwillingness to listen to the patient. |
| **11.4** | **Religious/cultural beliefs** | Patient is seen as difficult because of religious or cultural beliefs and practices that conflict with recommended care (e.g., refusing procedures or treatments). |
| **11.5** | **Social, economic and education level** | Patient is seen as difficult because of her social, economic or educational status, including poverty and limited ability to understand information given by providers. |
| **11.6** | **Use of TBAs** | Patient is seen as difficult because she came to the facility for delivery after first visiting a traditional birth attendant (TBA). |
| **11.7** | **When patient is provider** | Patient is seen as difficult because she is herself a health provider and is perceived to know a lot about medications or procedures, and may question or challenge care. |
| **11.8** | **Women empowerment** | Patient is seen as difficult because she knows her rights and asserts them, for example by asking questions, making demands or complaining. |
| **11.9** | **Women not following instruction/Uncooperative** | Patient is seen as difficult because she does not follow instructions or does the opposite of what providers ask. |
| **11.10** | **Women want to deliver on the floor** | Patient is seen as uncooperative because she prefers to deliver on the floor rather than on the hospital bed. |
| **12** | **Source of differential treatment** | Providers' perceptions of what causes providers to treat some patients differently from others, and examples of such differential treatment. Apply the relevant sub-code for each reason mentioned. |
| **12.1** | **Type of disease/prioritising emergencies** | Patients are treated differently or prioritised based on their condition, such as the type or severity of illness or the need for emergency care. |
| **12.2** | **Age** | Patients are treated differently based on their age (e.g., adolescents or older women). |
| **12.3** | **Connections/social ties with influential people** | Patients are treated differently because they have connections or social ties to influential people (e.g., politicians, senior staff, community leaders). |
| **12.4** | **Does not treat patients differently/no preferred patients** | Provider states that they (or providers at their facility) do not treat patients differently and have no preferred patients. |
| **12.5** | **Education/Wealth/socio-economic status** | Patients are treated differently based on education, wealth or socio-economic status. |
| **12.6** | **Gender** | Patients or their companions are treated differently based on gender. |
| **12.7** | **Occupation** | Patients are treated differently based on their occupation, including giving different treatment to nurses or other health workers. |
| **12.8** | **Parity** | Patients are treated differently based on the number of children they have had (parity), e.g., preference for first-time mothers (primigravidae) compared with women who have given birth before (multigravidae), or vice versa. |
| **12.9** | **Patient appearance** | Patients are treated differently based on how they look, such as their dress, cleanliness or personal hygiene. |
| **12.10** | **Patient attitude** | Patients are treated differently based on their attitude or behaviour, including lack of cooperation. |
| **12.11** | **Patient understanding** | Patients are treated differently based on how well they understand information and instructions from providers. |
| **12.12** | **Personal connections** | Patients are treated differently because they have a personal relationship with the provider (e.g., relative, friend, neighbour, acquaintance). |
| **12.13** | **Prior TBA birth** | Patients are treated differently because they first gave birth or sought care with a traditional birth attendant (TBA) before coming to the facility. |
| **12.14** | **Religion** | Patients are treated differently based on their religion. |
| **12.15** | **Stress or burnout** | Differential treatment is attributed to the provider's own stress or burnout rather than to patient characteristics. |
| **12.16** | **Tribe** | Patients are treated differently based on their tribe or ethnic group. |
| **12.17** | **Women's knowledge of their rights** | Patients are treated differently based on how aware they are of their rights (e.g., women who know their rights receive better or worse treatment). |
| **13** | **Source of satisfaction** | Responses to "What makes you most satisfied or happy about your job?" and other descriptions of what gives the provider satisfaction at work. |
| **13.1** | **Alleviating pain** | Provider finds satisfaction in relieving patients' pain or suffering. |
| **13.2** | **Being able to work without interference** | Provider finds satisfaction in being able to do their work independently, without interference from superiors, colleagues or patients' families. |
| **13.3** | **Feeling appreciated** | Provider finds satisfaction in being appreciated by others (e.g., superiors, colleagues, community) for their work. |
| **13.4** | **Feeling knowledgeable/capable of doing their job** | Provider finds satisfaction in feeling knowledgeable, skilled and capable of doing their job well. |
| **13.5** | **Feeling of being able to help** | Provider finds satisfaction in being able to help people. |
| **13.6** | **Feeling of doing your best** | Provider finds satisfaction in knowing they have done their best, regardless of the outcome. |
| **13.7** | **Feeling valued by patients** | Provider finds satisfaction when patients show that they value or appreciate them (e.g., thanking them, returning to them, recommending them). |
| **13.8** | **Financial incentives** | Provider finds satisfaction in financial rewards such as salary, allowances, bonuses or other incentives. |
| **13.9** | **Opportunity for growth** | Provider finds satisfaction in opportunities to learn, grow and advance professionally (e.g., training, promotion). |
| **13.10** | **Promoting life** | Provider finds satisfaction in saving or bringing new life, such as helping mothers and babies survive. |
| **13.11** | **Successful delivery** | Provider finds satisfaction in a delivery going well, with a healthy mother and baby. |
| **13.12** | **Successful resuscitation** | Provider finds satisfaction in successfully resuscitating a baby or patient. |
| **13.13** | **When patient is satisfied/happy** | Provider finds satisfaction when patients are satisfied or happy with the care they received. |
| **14** | **Stress** | Broad code for all data about stress experienced by providers, including its causes and how providers cope. Apply the relevant child codes. |
| **14.1** | **Causes of stress** | Provider descriptions of the factors that cause them stress. Apply the relevant sub-code for work-related or non-work-related causes. |
| 14.1.1 | Non-work related stress | Stress arising from outside work, including personal and family problems. |
| 14.1.1.1 | Financial strain | Stress caused by financial difficulties, such as debts, family financial obligations or not having enough money. |
| 14.1.1.2 | Loss of family member | Stress caused by the death of a family member. |
| 14.1.2 | Traumatic experience | Narratives of traumatic experiences that caused the provider significant distress. Can be double coded with Worst day (16.3). |
| 14.1.2.1 | Playing roles they are not trained for | Stress caused by having to perform roles or procedures the provider has not been trained for (e.g., due to staff shortages). |
| 14.1.3 | Work related source of stress | Stress arising from the provider's work, including workload, lack of supplies, patient and family behaviours, and relationships with colleagues and management. Apply the relevant sub-code. |
| 14.1.3.1 | Attitude and behavior of patients/families | Stress caused by the attitudes and behaviours of patients or their families, e.g., not following instructions, not cooperating, being rude or aggressive. |
| 14.1.3.2 | Baby needing resuscitation | Stress caused by having to resuscitate a newborn. |
| 14.1.3.3 | Community beliefs | Stress caused by community beliefs or practices that affect care, such as not preparing for delivery or distrust of the facility. |
| 14.1.3.4 | Death of a patient | Stress caused by the death of a patient, including a mother or baby. |
| 14.1.3.5 | Delayed or inadequate salary | Stress caused by salaries that are paid late, not paid, or too low. |
| 14.1.3.6 | Delayed or no promotion | Stress caused by delayed promotion or not being promoted. |
| 14.1.3.7 | Differential treatment from supervisors | Stress caused by supervisors or managers treating some staff more favourably than others. |
| 14.1.3.8 | Fear of losing a baby or mother | Stress caused by fear or worry that a baby or mother might die, including how the provider felt about this possibility. |
| 14.1.3.9 | Inadequate knowledge | Stress caused by the provider feeling they lack the knowledge or skills needed to manage a situation. |
| 14.1.3.10 | Inadequate staff/High patient volume/High work loads | Stress caused by too few staff, high patient numbers or heavy workloads. |
| 14.1.3.11 | Job security | Stress caused by uncertainty about keeping their job (e.g., contract staff, threat of transfer or dismissal). |
| 14.1.3.12 | Lack of drugs, equipment, and supplies | Stress caused by shortages of medicines, equipment or supplies needed to provide care. |
| 14.1.3.13 | Lack of personal time | Stress caused by not having enough time for personal life, rest or family because of work. |
| 14.1.3.14 | Low reward compared to effort | Stress caused by feeling that the pay or recognition received is too low for the effort put in. |
| 14.1.3.15 | Negative feedback from colleagues and superiors | Stress caused by criticism, blame or negative comments from colleagues or superiors. |
| 14.1.3.16 | Not being able to meet patient needs | Stress caused by being unable to meet patients' needs or provide the care they need (e.g., due to lack of resources). |
| 14.1.3.17 | Not feeling appreciated | Stress caused by feeling that their work is not appreciated by patients, colleagues, superiors or the health system. |
| 14.1.3.18 | Other providers not doing work well | Stress caused by other providers not doing their work properly, including mismanagement of a case by another provider. |
| 14.1.3.19 | Poor labor progress | Stress caused by a woman's labour not progressing as fast as expected. |
| 14.1.3.20 | Prolonged work hours | Stress caused by working long hours or extended shifts. |
| 14.1.3.21 | Satisfied with reward | Provider states they are satisfied with their pay or rewards, so this is not a source of stress for them. |
| 14.1.3.22 | Unique experiences of contract | Stress caused by experiences specific to being employed on a contract basis (e.g., lower pay, fewer benefits, job insecurity). |
| 14.1.3.23 | Unsupportive leadership style of superiors | Stress caused by superiors' leadership or supervision style, including complaints about unsupportive, harsh or unfair supervisors. |
| 14.1.3.24 | Unsupportive or lack of responsible behavior by other providers | Stress caused by other providers being unsupportive or not taking responsibility (e.g., not helping, leaving work for others, lateness). |
| 14.1.3.25 | When other staff are absent | Stress caused by colleagues being absent, leaving the provider to cover extra work. |
| 14.1.3.26 | When the patient is a provider | Stress caused by caring for a patient who is herself a health provider (e.g., fear of being judged or questioned). |
| 14.1.3.27 | Women going first to TBAs or giving birth at home | Stress caused by women going to traditional birth attendants (TBAs) first or giving birth at home, including complaints about complications in the woman or baby because she was given herbs by a TBA or chose to give birth at home. |
| 14.1.3.28 | Women presenting late in labor | Stress caused by women arriving at the facility late in labour. |
| 14.1.3.29 | Women presenting without required items | Stress caused by women arriving at the facility without the items they are expected to bring for delivery (e.g., pads, cloths, baby clothes). |
| **14.2** | **Coping strategies** | What the provider does to manage or reduce stress. Apply the relevant sub-code for each strategy mentioned. |
| 14.2.1 | Ask for help | Provider copes by asking colleagues, superiors, friends or family for help or support. |
| 14.2.2 | Do something else to distract | Provider copes by doing another activity to take their mind off the stress (e.g., farming or digging, cooking, going for a walk). |
| 14.2.3 | Drink alcohol | Provider copes by drinking alcohol. |
| 14.2.4 | Drink water | Provider copes by drinking water, often to calm down in the moment. |
| 14.2.5 | Keeping to self | Provider copes by withdrawing, keeping to themselves or staying quiet rather than sharing their feelings. |
| 14.2.6 | Listen to music/sing | Provider copes by listening to music or singing. |
| 14.2.7 | Not acknowledging stress as an issue | Provider does not see stress as an issue, denies experiencing stress, or says they simply ignore it. |
| 14.2.8 | Pray/go to church | Provider copes by praying or going to church. |
| 14.2.9 | Rationalization | Provider copes by trying to make sense of or justify a stressful event (e.g., "it was God's will", "I did all I could"). |
| 14.2.10 | Step away to cool down | Provider copes by briefly stepping away from the situation to calm down before returning. |
| 14.2.11 | Taking time to relax or sleep | Provider copes by taking time to rest, relax or sleep. |
| 14.2.12 | Talking with friends and family | Provider copes by talking with friends or family about their stress or problems. |
| 14.2.13 | Transfer of staff | Provider copes by requesting or seeking a transfer to another unit or facility, or describes staff transfer as a way to relieve stress. |
| 14.2.14 | Use of phone | Provider copes by using their phone (e.g., social media, calling someone, games). |
| 14.2.15 | Watch TV or movies | Provider copes by watching TV or movies. |
| **15** | **Training/Support/Mentorship** | Discussions of training, mentorship, peer support and other forms of support for providers, both past experiences and future needs. Apply the relevant child codes. |
| **15.1** | **Past/current support** | Descriptions of support the provider has received or currently receives, and its benefits, including peer support. |
| **15.2** | **Future mentorship** | Provider's needs, desires or preferences for mentorship in the future (e.g., who should mentor them, how it should work). |
| **15.3** | **Future support** | Provider's needs, desires or preferences for support in the future. Apply the relevant sub-code for the type of support. |
| 15.3.1 | Addressing challenges | Support that addresses the challenges providers face, such as providing equipment and supplies or adding more staff. |
| 15.3.2 | Cordial working relations | Support in the form of better, friendlier and more respectful working relationships among staff and with superiors. |
| 15.3.3 | Counseling | Support in the form of counselling or psychological support for providers. |
| 15.3.4 | Increase salary | Support in the form of higher pay. |
| 15.3.5 | Job security | Support in the form of more secure employment (e.g., permanent positions rather than contracts). |
| 15.3.6 | Motivation | Support that motivates providers, including incentives and the provision of food and drink. |
| 15.3.6.1 | Providing tea and other refreshment | Specifically, providing tea, snacks or other refreshments for staff. |
| 15.3.7 | Personal time off | Support in the form of time off, leave or breaks for rest and personal matters. |
| 15.3.8 | Recognition | Support in the form of recognition for good work (e.g., awards, praise, acknowledgement by management). |
| 15.3.9 | Team building activities | Support in the form of team-building activities to strengthen relationships among staff. |
| 15.3.10 | Training/continuous education | Support in the form of training and continuing professional education. |
| **15.4** | **Future training** | Providers' needs and preferences for future training, for themselves or other staff. Apply the relevant sub-code for content, format, length, location or participants. |
| 15.4.1 | Content | The topics that providers would like future training to cover. |
| 15.4.1.1 | Clinical knowledge | Training on clinical knowledge and skills (e.g., emergency obstetric care, newborn resuscitation). |
| 15.4.1.2 | Dealing with different emotions | Training on recognising and managing different emotions, their own and others'. |
| 15.4.1.3 | Handling negative feedback | Training on how to receive and respond to criticism or negative feedback. |
| 15.4.1.4 | How to motivate others | Training on how to motivate colleagues or staff. |
| 15.4.1.5 | Interactions with patients | Training on communicating and interacting with patients (e.g., respectful care, communication skills). |
| 15.4.1.6 | Managing stress | Training on how to manage or cope with stress. |
| 15.4.2 | Format | How providers would like future training delivered, including group or individual, in person or virtual, types of participants, who delivers it and where. |
| 15.4.2.1 | Digital | Preference for training delivered digitally or virtually (e.g., online, via phone or app). |
| 15.4.2.2 | Group | Preference for training delivered in a group. |
| 15.4.2.3 | Individual | Preference for training delivered individually or one-on-one. |
| 15.4.2.4 | No digital | Preference for training that is not digital, i.e., delivered in person. |
| 15.4.3 | Length | Preferences for how long training should last (e.g., number of days, hours per session, frequency). |
| 15.4.4 | Meditation | Suggestions or preferences for meditation or relaxation practices as part of training. |
| 15.4.5 | Where | Preferences for where training should take place (e.g., at the facility, off-site, at a district or regional location). |
| 15.4.6 | Who | Preferences for who should deliver the training and who should attend or be included. |
| 15.4.6.1 | All cadres of providers including support staff | Preference for training to include all cadres of providers, including support staff, together. |
| 15.4.6.2 | Support staff separately | Preference for support staff to be trained separately from clinical providers. |
| **15.5** | **Past training** | Any discussion of training the provider has received in the past. |
| 15.5.1 | Differential selection for training | Provider describes unfair or unequal selection of who gets to attend training (e.g., favouritism). |
| **15.6** | **Past/current mentorship** | Descriptions of the provider's past or current experiences of mentorship, as mentor or mentee. |
| 15.6.1 | Non-work mentors/mentees | Mentorship relationships outside of work (e.g., mentors or mentees from church, family or community). |
| 15.6.2 | Relationship with mentor | Descriptions of the provider's relationship with their mentor (e.g., quality, trust, frequency of contact). |
| **15.7** | **Peer support** | Discussions of support from fellow providers at a similar level, and views on peer support. Apply the relevant sub-code for their preference. |
| 15.7.1 | Does not want peer support | Provider does not want peer support or expresses reservations about it (e.g., concerns about confidentiality or trust). |
| 15.7.2 | Would like peer support | Provider would like to have peer support or expresses interest in it. |
| **16** | **Work day** | Descriptions of the provider's work day at the facility, including typical, best and worst days. Apply the relevant child codes. |
| **16.1** | **Best day** | Narratives of the provider's best day at work. Apply the relevant sub-code for what made it the best day. |
| 16.1.1 | Financial incentive | Best day was because the provider received money, a bonus or another financial reward. |
| 16.1.2 | No complications | Best day was because deliveries or cases went smoothly without complications. |
| 16.1.3 | Non-work related best day | Provider describes a best day that is not related to work (e.g., a personal or family event). |
| 16.1.4 | Successful management of a difficult situation | Provider describes a difficult situation as their best day because they handled it well and it had a good outcome. |
| **16.2** | **Typical day** | Descriptions of the provider's typical day at work, in response to the general question "Tell me what a typical work day looks like for you", including routines, tasks, shifts and workload. |
| **16.3** | **Worst day** | Descriptions of the provider's worst day at work, including what happened, why, and how they felt. Apply the relevant sub-code for what made it the worst day. |
| 16.3.1 | Boring because of few deliveries | Worst day was because there were few or no deliveries, making the day boring. |
| 16.3.2 | Death of a patient | Worst day involved the death of a patient (mother or baby). |
| 16.3.3 | Difficult case | Worst day involved a clinically difficult or complicated case. |
| 16.3.4 | High work load | Worst day involved a very heavy workload or large number of patients. |
| 16.3.5 | Litigation | Worst day involved legal action, or the threat of legal action, against the provider or facility. |
| 16.3.6 | Mismanagement of case | Worst day involved a case being mismanaged, by the provider or others. |
| 16.3.7 | Patient developed complications | Worst day involved a patient developing complications. |
| 16.3.8 | Personal relationship with client or fellow provider | Worst day involved a conflict or problem in the provider's personal relationship with a patient or a fellow provider. |
| 16.3.9 | Punished for something you did not do | Worst day involved the provider being blamed or punished for something they did not do. |
| 16.3.10 | Traumatic experience | Descriptions of traumatic experiences at work that the provider identifies as a worst day. Can be double coded with Traumatic experience (14.1.2). |
