# Supplementary material for: Power, autonomy, and the construction of the ‘difficult patient’ in the labor ward: Insights from maternal health providers in western Kenya and northern Ghana
Source: PLOS Glob Public Health. 2026 Nov 13;6(11):e0007069. doi: 10.1371/journal.pgph.0007069 (PMC13766216; doi:10.1371/journal.pgph.0007069)
Supplement: Supplementary file 5 [file pgph.0007069.s005.docx]

September 30, 2026

The Editor

PLOS Global Public Health

**RE: Response letter**

We would like to express our gratitude to the reviewers for their timely and thorough review of our manuscript. We hereby provide detailed responses to the reviewers' comments provided below. A clean version of the paper is included in this resubmission, along with the version with tracked changes. We are confident the changes have resulted in a strengthened manuscript.

Sincerely,

Patience Afulani

On behalf of the study team

| **Reviewer #1 Comments** | **Response** |
| --- | --- |
| I appreciate the opportunity to review this manuscript analyzing factors associated with labeling of a ‘difficult’ patient, a commonly encountered situation across many healthcare contexts but particularly among laboring patients. The introduction provided excellent context for the rationale behind the study, particularly as to why the particular regions of study were selected. I appreciated the clearly defined research questions, and feel that the results section did a very good job of answering these questions and the discussion highlighted key implications of this work. However, there are some gaps in the methods section that require clarification. | Thank you for the kind and supportive comments. We have addressed the gaps noted |
| The data collection section leaves me with some questions. First, it states that “data procedures are described in detail elsewhere,” but it is unclear where these would be found. It would be helpful to describe these procedures more or to include a supplemental material that has them. | Thank you for your comment.  The citations provided are where the study procedures have been described. We however acknowledge readers are unlikely to review the methods in prior papers. We have thus provided additional relevant details on the data collection procedures in the manuscript as well as cited the prior papers from the same study that also describe the methods  See line: 120-129 |
| In addition, you describe the timeframe that interviews occurred, but there is no explanation for the significant time gap between 2019 and 2021. | Thank you for the observation. The study was initially conducted in Kenya and was subsequently replicated in Ghana, with some delays in Ghana during the early period of the COVID-19 pandemic We have revised the text to clearly indicate this.  See line: 137-140 |
| I would also appreciate details into how facilities were selected and how recruitment was done at the selected facilities. | We are happy to include the details as per your request. We have now included how facilities were selected and how recruitment was done at the selected facilities in the data collection section.  See line 120-132 |
| Later in the paper, you acknowledge that no men from Ghana were included, but I would appreciate an explanation as to why this occurred? | Thank you for the observation. The absence of male participants from Ghana in this study is due to the staffing composition of maternity wards in Ghana, where the majority of the providers are midwives, and the midwifery profession in Ghana is predominantly female. This results from cultural and gender-sensitive influences rather than from male midwifery professional competences. We have noted this in the limitations (since we can’t discuss in the results section).  See lines 663-655 |
| Finally, the data analysis section was very clear and thorough for the interview analysis, but what was done with the information from the surveys that the providers completed? | Thanks for the comment and we acknowledge the lack of clarity. We have now clarified that this study was nested within a larger study addressing several questions to inform an intervention study.  Although the larger study was a mixed methods study, the findings have been presented in a series of papers answering specific research questions, some as mixed methods papers, and others as quantitative or qualitative papers. We have provided citations for these publications.  The data on the difficult situations were only included from the in-depth interviews, hence only qualitative results in this manuscript. In fact this paper is based on just a few questions in the interview guide which yielded great data.  See line 89-90, and 130-134. |
| There were several grammatical errors throughout, that although they did not detract from the readability of the manuscript, would be helpful to correct (i.e. Line 50, 51, 71-72, 101, 312, 318) along with the inconsistent capitalization in the title and italicization and decimals in Table 1. | Thank you for the careful read and picking up these errors. We have reviewed carefully and corrected all grammatical errors. For quotes, we only edit to increase readability, but try to maintain them as transcribed.  We revised the title for consistent capitalization and to simplify it to just one semi-colon  We have standardized italicization and decimal places in Table 1 (all percentages are now reported to one decimal place).  Please note that some final minor editorial edits are only reflected in the clean version. |
|  | Your thoughtful comments have strengthened the manuscript. Thank you |

| **Reviewer #2 Comments** | **Response** |
| --- | --- |
| Thank you for the opportunity to review this well-written manuscript looking at power, autonomy, and the construction of the ‘difficult patient’. I thought the reflexivity and discussion sections were particularly strong.  I have a few general suggestions, and some regarding the analysis and theme construction, as follows: | Thank you for the kind and supportive comments. We have incorporated your suggestions |
| P 4, line 115 – suggest rephrasing this to say the interviews lasted from 40-60 minutes – at the moment it reads as if it is the interview guides. | Thank you. The sentence now reads: "The interviews lasted from 40–60 minutes…"  See Line 137 |
| Line 120 – the reference given for codebook analysis (30) is a systematic review, not a methodology paper, please correct. | Thank you for this keen observation, we have corrected this and reviewed all the references to ensure that they reflect correctly.  Code book analysis ref now 35 by Braun and Clark where they describe different types of thematic analysis.  See line 144 |
| The subtheme ‘patient’s psychological state’ is a little thin – I suggest merging it with the theme above, which contains all of the patient characteristics that providers seem to think lead to difficult situations. It was good to see this idea unpacked in the discussion. | Thank you for this suggestion. We agree that the sub-theme “patient’s psychological state’ is a little thin and can be merged with the theme above. So we have done that and renamed that theme to be inclusive of this subtheme.  See lines 185-188 and 298-299. |
| I think ‘use of herbal medications’ would fit better under the Patient’s beliefs and preferences theme | Thank you. We considered this carefully, and we think it could fit under either theme. But given providers described herbal medication use primarily in terms of the behavior and its clinical consequences rather than as a matter of belief, we have kept it under patients' behaviors to maintain that emphasis.  See line 264-268-259 |
| The theme ‘Challenging Workplace Conditions’ also needs a bit more in it to be a theme in itself. Is there a bit more data and analysis you can add here? | Thank you. Yes, there is more data. We have expanded this theme with the additional data to highlight how not having certain resources and capabilities contributed to difficult situations. We have also strengthened other themes that appeared thin with additional data.  See lines 368-392 |
| Overall, the paper makes a useful contribution to the literature | Thank you very much. We appreciate all your suggestions to strengthen the paper |

**Comments from the editorial office**

| **Editor Comments** | **Response** |
| --- | --- |
| The reviewers and I found the manuscript to be well-written and offer helpful insights into how maternal healthcare providers and staff, as well as structural and social conditions, contribute to disrespect and abuse during childbirth. There are just a few areas for clarification and minor edits that will further strengthen the manuscript.  In addition to the reviewers' questions, I would encourage the authors to provide the final interview guide and codebook as supplemental materials. | We appreciate the support for this work. We have now included the final interview guide and codebook as supplemental materials (S1 and S2). |
| **Journal Requirements:** | **Response** |
| 1.Please include a complete copy of PLOS’ questionnaire on inclusivity in global research in your revised manuscript | The completed PLOS inclusivity in global research questionnaire is provided as S3 File |
| 2. In the online submission form, you indicated that “All relevant data are reported within the manuscript. Additional data can be obtained from the corresponding author on reasonable request.”.  All PLOS journals now require all data underlying the findings described in their manuscript to be freely available to other researchers, either  1. In a public repository,  2. Within the manuscript itself, or  3. Uploaded as supplementary information.  This policy applies to all data except where public deposition would breach compliance with the protocol approved by your research ethics board. If your data cannot be made publicly available for ethical or legal reasons (e.g., public availability would compromise patient privacy), please explain your reasons by return email and your exemption request will be escalated to the editor for approval. Your exemption request will be handled independently and will not hold up the peer review process, but will need to be resolved should your manuscript be accepted for publication. One of the Editorial team will then be in touch if there are any issues. | We are unable to publicly share the individual transcripts because they contain identifying information about the providers.  Sharing transcripts publicly will also breach our ethics approval, which assured participants their data will only be shared in aggregate form and not individually. We considered uploading excerpts of the coded texts from dedoose as supplementary information, but these are all tagged with facility name and provider type which could be used to identify providers given the few providers in several of the facilities. Public deposition would therefore breach compliance with the protocol approved by our research ethics board.  We have however included the additional data underlying the analysis within the manuscript such that all relevant data underlying the findings described in the manuscript are within the manuscript. We have updated the Data Availability statement to note “All relevant data are reported within the manuscript”  We will also submit an exemption request by email as instructed. |
| 3. Please ensure that your Ethics Statement is available in its entirety at the beginning of your Methods section, under a subheading 'Ethics Statement'. | The Ethics Statement now appears at the start of the Methods under the heading "Ethics Statement"  See lines 95-102 |
| If the reviewer comments include a recommendation to cite specific previously published works, please review and evaluate these publications to determine whether they are relevant and should be cited. There is no requirement to cite these works unless the editor has indicated otherwise.   Please review your reference list to ensure that it is complete and correct. If you have cited papers that have been retracted, please include the rationale for doing so in the manuscript text, or remove these references and replace them with relevant current references. Any changes to the reference list should be mentioned in the rebuttal letter that accompanies your revised manuscript. If you need to cite a retracted article, indicate the article’s retracted status in the References list and also include a citation and full reference for the retraction notice. | We reviewed the reference list and have: replaced reference [32 now 35] with a the right source; merged duplicate references [14] and [36]; corrected the formatting of references [3, 9, 22, 25, 39, 44]. We have also added references [30-32] in response to the reviewer comment on where the quantitative studies are published.  No retracted works are cited. |
