# Supplementary material for: Power, autonomy, and the construction of the ‘difficult patient’ in the labor ward: Insights from maternal health providers in western Kenya and northern Ghana
Source: PLOS Glob Public Health. 2026 Nov 13;6(11):e0007069. doi: 10.1371/journal.pgph.0007069 (PMC13766216; doi:10.1371/journal.pgph.0007069)
Supplement: Peer review history

# Peer review: Decision Letter 0

**DOI:** 10.1371/journal.pgph.0007069.r001

**Authors:** Marie A. Brault

1 Sep 2026

PGPH-D-26-02886

Navigating the Labor Ward: Power, Autonomy, and the Construction of the ‘Difficult Patient’: Insights from maternal health providers from Western Kenya and Northern Ghana

PLOS Global Public Health

Dear Dr. Afulani,

Thank you for submitting your manuscript to PLOS Global Public Health. After careful consideration, we feel that it has merit but does not fully meet PLOS Global Public Health’s publication criteria as it currently stands. Therefore, we invite you to submit a revised version of the manuscript that addresses the points raised during the review process.

==============================

Editor comments:

- The reviewers and I found the manuscript to be well-written and offer helpful insights into how maternal healthcare providers and staff, as well as structural and social conditions, contribute to disrespect and abuse during childbirth. There are just a few areas for clarification and minor edits that will further strengthen the manuscript.
- In addition to the reviewers' questions, I would encourage the authors to provide the final interview guide and codebook as supplemental materials.

==============================

Please submit your revised manuscript by Oct 01 2026 11:59PM. If you will need more time than this to complete your revisions, please reply to this message or contact the journal office at globalpubhealth@plos.org. When you're ready to submit your revision, log on to https://www.editorialmanager.com/pgph/ and select the 'Submissions Needing Revision' folder to locate your manuscript file.

Please include the following items when submitting your revised manuscript:

- A letter that responds to each point raised by the editor and reviewer(s). You should upload this letter as a separate file labeled 'Response to Reviewers'.
- A marked-up copy of your manuscript that highlights changes made to the original version. You should upload this as a separate file labeled 'Revised Manuscript with Track Changes'.
- An unmarked version of your revised paper without tracked changes. You should upload this as a separate file labeled 'Manuscript'.

Guidelines for resubmitting your figure files are available below the reviewer comments at the end of this letter.

As the corresponding author, your ORCID iD is verified in the submission system and will appear in the published article. PLOS supports the use of ORCID, and we encourage all coauthors to register for an ORCID iD and use it as well. Please encourage your coauthors to verify their ORCID iD within the submission system before final acceptance, as unverified ORCID iDs will not appear in the published article. *Only*  the individual author can complete the verification step; PLOS staff *cannot*  verify ORCID iDs on behalf of authors.

We look forward to receiving your revised manuscript.

Kind regards,

Marie A. Brault, PhD

Academic Editor

PLOS Global Public Health

**Journal Requirements:**

1. Please include a complete copy of PLOS’ questionnaire on inclusivity in global research in your revised manuscript. Our policy for research in this area aims to improve transparency in the reporting of research performed outside of researchers’ own country or community. The policy applies to researchers who have travelled to a different country to conduct research, research with Indigenous populations or their lands, and research on cultural artefacts. The questionnaire can also be requested at the journal’s discretion for any other submissions, even if these conditions are not met.  Please find more information on the policy and a link to download a blank copy of the questionnaire here: https://journals.plos.org/globalpublichealth/s/best-practices-in-research-reporting. Please upload a completed version of your questionnaire as Supporting Information when you resubmit your manuscript.

2. In the online submission form, you indicated that “All relevant data are reported within the manuscript. Additional data can be obtained from the corresponding author on reasonable request.”.

All PLOS journals now require all data underlying the findings described in their manuscript to be freely available to other researchers, either

1. In a public repository,

2. Within the manuscript itself, or

3. Uploaded as supplementary information.

This policy applies to all data except where public deposition would breach compliance with the protocol approved by your research ethics board. If your data cannot be made publicly available for ethical or legal reasons (e.g., public availability would compromise patient privacy), please explain your reasons by return email and your exemption request will be escalated to the editor for approval. Your exemption request will be handled independently and will not hold up the peer review process, but will need to be resolved should your manuscript be accepted for publication. One of the Editorial team will then be in touch if there are any issues.

3. Please ensure that your Ethics Statement is available in its entirety at the beginning of your Methods section, under a subheading 'Ethics Statement'.

If the reviewer comments include a recommendation to cite specific previously published works, please review and evaluate these publications to determine whether they are relevant and should be cited. There is no requirement to cite these works unless the editor has indicated otherwise.

Please review your reference list to ensure that it is complete and correct. If you have cited papers that have been retracted, please include the rationale for doing so in the manuscript text, or remove these references and replace them with relevant current references. Any changes to the reference list should be mentioned in the rebuttal letter that accompanies your revised manuscript. If you need to cite a retracted article, indicate the article’s retracted status in the References list and also include a citation and full reference for the retraction notice.

**Additional Editor Comments (if provided):**

[Note: HTML markup is below. Please do not edit.]

Reviewers' comments:

Reviewer's Responses to Questions

**Comments to the Author**

1. Does this manuscript meet PLOS Global Public Health’s publication criteria?

Reviewer #1: Yes

Reviewer #2: Yes

**********

2. Has the statistical analysis been performed appropriately and rigorously?

Reviewer #1: Yes

Reviewer #2: N/A

**********

3. Have the authors made all data underlying the findings in their manuscript fully available (please refer to the Data Availability Statement at the start of the manuscript PDF file)?

The PLOS Data policy

Reviewer #1: Yes

Reviewer #2: No

**********

4. Is the manuscript presented in an intelligible fashion and written in standard English?

Reviewer #1: Yes

Reviewer #2: Yes

**********

**Reviewer #1** : I appreciate the opportunity to review this manuscript analyzing factors associated with labeling of a ‘difficult’ patient, a commonly encountered situation across many healthcare contexts but particularly among laboring patients. The introduction provided excellent context for the rationale behind the study, particularly as to why the particular regions of study were selected. I appreciated the clearly defined research questions, and feel that the results section did a very good job of answering these questions and the discussion highlighted key implications of this work. However, there are some gaps in the methods section that require clarification.

The data collection section leaves me with some questions. First, it states that “data procedures are described in detail elsewhere,” but it is unclear where these would be found. It would be helpful to describe these procedures more or to include a supplemental material that has them. In addition, you describe the timeframe that interviews occurred, but there is no explanation for the significant time gap between 2019 and 2021. I would also appreciate details into how facilities were selected and how recruitment was done at the selected facilities. Later in the paper, you acknowledge that no men from Ghana were included, but I would appreciate an explanation as to why this occurred. Finally, the data analysis section was very clear and thorough for the interview analysis, but what was done with the information from the surveys that the providers completed?

The results section is very well organized and this makes it easy for readers to follow. The direct quotes are excellent and support your themes well. I agree with the statement about the limitations in not addressing patients’ perspectives and think this is a strong next step for investigation.

There were several grammatical errors throughout, that although they did not detract from the readability of the manuscript, would be helpful to correct (i.e. Line 50, 51, 71-72, 101, 312, 318) along with the inconsistent capitalization in the title and italicization and decimals in Table 1.

**Reviewer #2:**  Thank you for the opportunity to review this well-written manuscript looking at power, autonomy, and the construction of the ‘difficult patient’. I thought the reflexivity and discussion sections were particularly strong.

I have a few general suggestions, and some regarding the analysis and theme construction, as follows:

P 4, line 115 – suggest rephrasing this to say the interviews lasted from 40-60 minutes – at the moment it reads as if it is the interview guides.

Line 120 – the reference given for codebook analysis (30) is a systematic review, not a methodology paper, please correct.

The subtheme ‘patient’s psychological state’ is a little thin – I suggest merging it with the theme above, which contains all of the patient characteristics that providers seem to think lead to difficult situations. It was good to see this idea unpacked in the discussion.

I think ‘use of herbal medications’ would fit better under the Patient’s beliefs and preferences theme.

The theme ‘Challenging Workplace Conditions’ also needs a bit more in it to be a theme in itself. Is there a bit more data and analysis you can add here?

Overall, the paper makes a useful contribution to the literature.

**********

what does this mean?). If published, this will include your full peer review and any attached files.

**Do you want your identity to be public for this peer review?**  If you choose “no”, your identity will remain anonymous but your review may still be made public.

For information about this choice, including consent withdrawal, please see our Privacy Policy

Reviewer #1: No

Reviewer #2: No

**********

[NOTE: If reviewer comments were submitted as an attachment file, they will be attached to this email and accessible via the submission site. Please log into your account, locate the manuscript record, and check for the action link "View Attachments". If this link does not appear, there are no attachment files.]

**Figure Resubmissions:**

# Author comment: Author response to Decision Letter 1

**DOI:** 10.1371/journal.pgph.0007069.r002

30 Sep 2026

Attachment Submitted filename: DS paper response to reviewer comments-9-30-26.docx

# Peer review: Decision Letter 1

**DOI:** 10.1371/journal.pgph.0007069.r003

**Authors:** Marie A. Brault

29 Oct 2026

Power, Autonomy, and the Construction of the ‘Difficult Patient’ in the Labor Ward: Insights from Maternal Health Providers in Western Kenya and Northern Ghana

PGPH-D-26-02886R1

Dear Dr. Afulani,

We are pleased to inform you that your manuscript 'Power, Autonomy, and the Construction of the ‘Difficult Patient’ in the Labor Ward: Insights from Maternal Health Providers in Western Kenya and Northern Ghana' has been provisionally accepted for publication in PLOS Global Public Health.

Before your manuscript can be formally accepted you will need to complete some formatting changes, which you will receive in a follow up email. A member of our team will be in touch with a set of requests.

Please note that your manuscript will not be scheduled for publication until you have made the required changes, so a swift response is appreciated.

IMPORTANT: The editorial review process is now complete. PLOS will only permit corrections to spelling, formatting or significant scientific errors from this point onwards. Requests for major changes, or any which affect the scientific understanding of your work, will cause delays to the publication date of your manuscript.

If your institution or institutions have a press office, please notify them about your upcoming paper to help maximize its impact. If they'll be preparing press materials, please inform our press team as soon as possible -- no later than 48 hours after receiving the formal acceptance. Your manuscript will remain under strict press embargo until 2 pm Eastern Time on the date of publication. For more information, please contact globalpubhealth@plos.org.

Thank you again for supporting Open Access publishing; we are looking forward to publishing your work in PLOS Global Public Health.

Best regards,

Marie A. Brault, PhD

Academic Editor

PLOS Global Public Health

***********************************************************

Reviewer Comments (if any, and for reference):

Reviewer's Responses to Questions

**Comments to the Author**

Reviewer #1: All comments have been addressed

**********

publication criteria?

Reviewer #1: Yes

**********

3. Has the statistical analysis been performed appropriately and rigorously?

Reviewer #1: N/A

**********

4. Have the authors made all data underlying the findings in their manuscript fully available (please refer to the Data Availability Statement at the start of the manuscript PDF file)?

The PLOS Data policy

Reviewer #1: Yes

**********

5. Is the manuscript presented in an intelligible fashion and written in standard English?

Reviewer #1: Yes

**********

Reviewer #1: (No Response)

**********

what does this mean?). If published, this will include your full peer review and any attached files.

**Do you want your identity to be public for this peer review?**  If you choose “no”, your identity will remain anonymous but your review may still be made public.

For information about this choice, including consent withdrawal, please see our Privacy Policy

Reviewer #1: No

**********
